# Primary Mucinous Adenocarcinoma of the Upper Eyelid in an African American Female: A Rare Clinical Entity. A Case Report and Literature Review

**DOI:** 10.7759/cureus.6254

**Published:** 2019-11-28

**Authors:** Prashanth Ashok Kumar, Shweta Paulraj, Seung S Hahn, Abirami Sivapiragasam

**Affiliations:** 1 Internal Medicine, Upstate Medical University, Syracuse, USA; 2 Radiation Oncology, Upstate Medical University, Syracuse, USA; 3 Hematology-Oncology, Upstate Medical University, Syracuse, USA

**Keywords:** primary mucinous adenocarcinoma, african-american, eyelid malignancy, mohs micrographic surgery, ck 7, ck 20, p63

## Abstract

Primary mucinous adenocarcinoma (PMA) of the eyelid is a rare eccrine gland cancer with an incidence of 0.07 per million person-years. We report a case of a 62-year-old African American female who presented with a tender lesion over her left upper eyelid which was gradually progressive over four years. It was initially presumed to be benign but histopathology after excision was suggestive of a mucinous colloid carcinoma with positive margins. She underwent repeat excision with wide margins and reconstruction and immunohistochemical studies were suggestive of PMA. Workup for metastatic disease and rare possibility of underlying occult malignancy was negative. PMA is uncommon, more so in the African American population and in females. Given the uncommon occurrence of this tumor and similarities in histopathology to colon and breast cancers, underlying occult malignancies need to be ruled out prior to confirming the diagnosis of PMA. The most effective treatment modalities are Mohs micrographic surgery or excision with frozen section control of margins with regular follow up over a prolonged period of time. However, there are no large clinical studies with regard to treatment and follow up. More literature on this tumor would therefore be beneficial to clinicians.

## Introduction

Primary mucinous adenocarcinoma (PMA) of the eyelid is an extremely uncommon group of cutaneous cancers that arise from the eccrine glands [1]. The first such tumor was reported by Lennox et al. way back in 1952 as a mucin secreting tumor of the skin [2]. Mendoza and Helwig were the first in 1971 to describe it as “Mucinous adenocystic carcinoma” [3]. It is safe to say that these tumors are most frequently noted in the head and neck region. Among around 200 reported cases of PMA of the skin, the eyelid by far remains the single most common area where it has been reported with a share of 41% [3-5]. PMA of the eyelid seems to occur twice as common in men than in women. It is more common in Caucasians with an average age of presentation being 62 years [6]. Although it has low rates of metastasis, the local recurrence rate is high. These tumors have unique immunohistochemical pathological features that play an important role in their diagnosis and in the distinction from a metastatic lesion [1, 6]. We report the case of a 62-year-old female with PMA of the left upper eyelid, its diagnosis, management, and subsequent follow up.

## Case presentation

The patient was a 62-year-old female of African American ethnicity, with a past medical history of type 2 diabetes mellitus, chronic kidney disease secondary to diabetes, glaucoma, macrocytic anemia, chronic back pain and hypertension, who initially presented to her ophthalmologist with a small growth over her left upper eyelid. The patient had the swelling for more than four years that had gradually increased in size. She did not have it evaluated as it very occasionally caused symptoms like on and off tearing, itching, and irritation. She decided to seek medical care when she noticed some tenderness over the lesion. On initial evaluation, she was noted to have a 0.6 mm, elevated, cystic-appearing subcutaneous nodule in the central medial left upper lid margin and lash row. It was noninflamed and tender to palpation. The lesion was in close proximity to the cornea. All other eyelid and orbital functions were normal.

**Figure 1:**
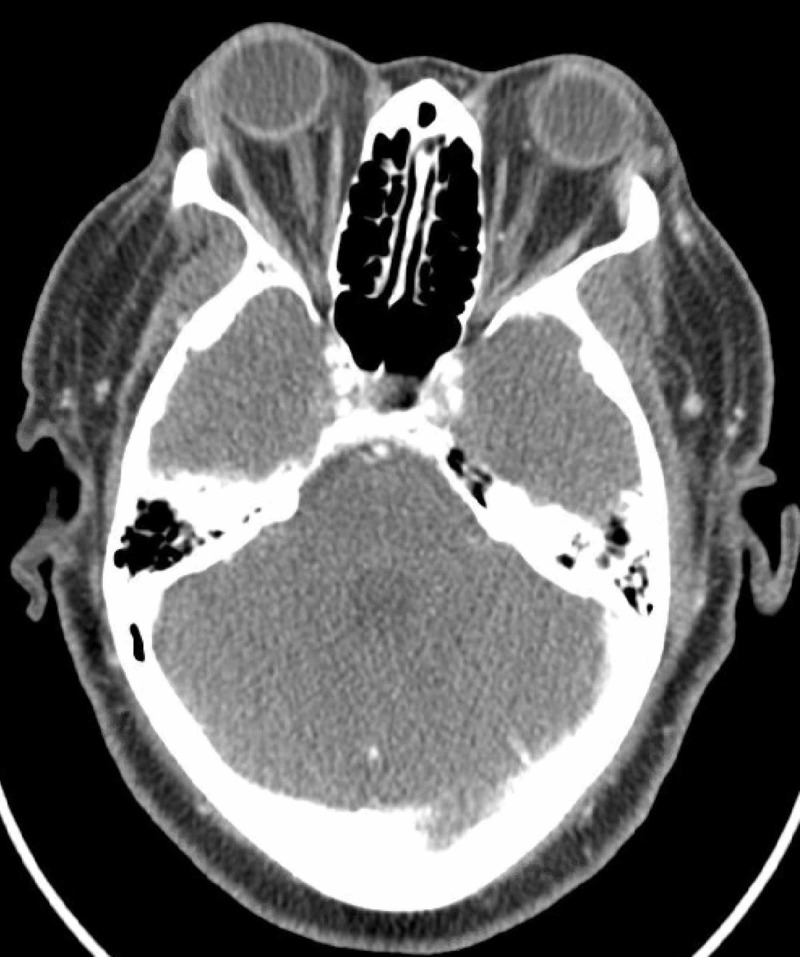
CT imaging of the orbit showing no residual or loco-regional extension of the lesion.

The lesion was initially thought to be a benign cyst. As it was symptomatic, it was excised and reconstruction was done. Histopathological analysis revealed positivity for carcinoma cells and suggested that it was a mucinous colloid carcinoma. As the margins were positive, a repeat excision with wider margins and reconstruction via advancement of her lateral full thickness upper lip into the central upper lid was done following a superior cantholysis of the lateral canthal tendon. The surgeons felt that there was no evidence of regional extension beyond what was excised. This was further confirmed by CT imaging of the orbits that revealed no locoregional extension or residual tumor (Figure 1). Histopathological exam showed that the tumor was present within the dermis abutting the orbicularis muscle and was composed of lobules of epithelial cells floating in pools of mucin. Small ductal structures were observed within the lobules. A dual population of epithelial cells was identified with mild to moderate pleomorphism admixed with some atypical mitotic figures. Adjacent to the tumor, there were distended ductal structures with atypical proliferation of epithelial cells with a cribriform architecture and extracellular mucin. The tumor extended into the deep tissue edges, but perineural invasion was not identified. Immunohistochemical analysis revealed that the tumor was positive for tumor protein p63 (P63) and cytokeratin 7 (CK7) and negative for cytokeratin 20 (CK20), thyroid transcription factor-1 (TTF-1), and human epidermal growth factor receptor 2 (HER-2). These findings confirmed the diagnosis of PMA of the eyelid.

Given that an underlying occult malignancy of the gastrointestinal tract or breast may present similarly, she was evaluated extensively with CT scans of the thorax, abdomen, and soft tissues of the neck. She also had a mammogram and a colonoscopy. All the tests were unremarkable and negative for malignancy. Given the rarity of the disease, Radiation Oncology and Medical Oncology services were integrated to her care. She is being followed up in regular intervals and has not had any recurrence of her tumor thus far.

## Discussion

With an incidence of 0.07 per million person-years and only around 80 cases so far, PMA of the eyelid is referred to by several names such as adenoid cystic carcinoma, mucinous carcinoma, mucoepidermoid carcinoma, mucin secreting carcinoma, and gelatinous carcinoma [7]. Most of the information on this rare tumor has been obtained from individual case reports [1, 7]. PMA of the eyelid remains to be a rare diagnosis as it is very commonly thought to be a benign swelling [1]. They have a very indolent and slow rate of growth which leads to delays in being brought to medical attention. The slow rate of growth seems to be attributed to the abundant mucin production by these tumors, which hamper and inhibit nutrition of the tumor cells [8]. The presentation of these tumors is varied and nonspecific and do not contribute much towards diagnosis. They usually appear as solitary, smooth, round, painless, papular or nodular lesions, ranging in size from 0.7 to 120 cm, with the average size being around 1.8 cm. The lesions are well circumscribed, unencapsulated, and sometimes may be stuck to the dermis [9]. Many infectious, inflammatory, and malignant mesenchymal and epithelial lesions have similar presentation and hence needs to be differentiated from PMA. Common differentials to be considered include lipoma, chalazion, epidermoid cyst, papilloma, keratocanthoma, nevus, apocrine histiocytoma, hemangioma, kaposi sarcoma, pyogenic granuloma, sebaceous cyst or carcinoma, squamous cell carcinoma (SCC), basal cell carcinoma (BCC), malignant melanoma (MM) and metastasis from other primary adenocarcinoma, which possibly is the most important differential [3].

The first large case series of the eyelid lesion was reported by Mendoza and Helwig who described it with the contemporary name as described above [3]. The largest study to date comprising 21 cases of eyelid PMA was reported by Wright and Font who suggested that the tumor likely originated from secretory cells of the eccrine coil [10]. The World Health Organization classifies it under the category of malignant tumors with apocrine and eccrine differentiation [3]. Based on findings seen in electron microscopy and immunohistochemistry, most authors have originally believed it to be more eccrine in origin than apocrine [11]. In histochemical studies, it was seen that that PMA had a large phosphorylase activity, limited nonspecific esterase and phosphatase activity and was positive for a number of mitochondrial oxidative enzymes, similar to eccrine cells. The appearance of PMA cells was also similar to dark cells of the normal eccrine glands. However, a few reports have shown certain apocrine ultrastructural features like vacuolization and electron dense bodies [12]. This has led to discussions questioning its description as an eccrine tumor, making the debate on whether it is eccrine or apocrine ongoing and unsettled [3].

Kamalpour et al. performed a meta-analysis of case reports of PMA after surgery from 1952 to 2010 to analyze the demographic characteristics, treatment outcomes, and prognostic factors. Of 159 cases of PMA, 49.7% were of the eyelid, eyebrows, or canthus. Demographic analysis showed that 77.2% were Caucasians, 12.7 % were East Asian and Indians, and only 10.1% were African Americans. Considering these factors, our case is a relatively rare presentation given our patient is a female of African American origin. Eyelid and other head and neck PMA had better outcomes when compared to PMA from other parts of the body like the trunk, which is opposite to that seen with SCC and BCC. Tumors with an initial size of > 1.5 cm had a higher rate of recurrence and metastasis. The main factor associated with mortality was incomplete resection of the tumor, leading to recurrence, which is generally more resistant to radiation therapy (RT) and chemotherapy (CT) [13].

The PMA of the eyelid is always diagnosed only by histopathological and immuohistochemical analysis [14]. The cells generally have a centrally placed, cuboidal nucleus, and an eosinophilic cytoplasm with some mitosis. The lesions are well differentiated given that they produce a lot of diastase resistant, periodic acid Schiff positive, hyaluronidase resistant and acidic mucin, which is a sign of synthetic function seen with mature cells. They are also avascular explaining a possible reason for the low metastasis potential [6]. Our case had similar findings in histopathology consistent with that seen in other mucinous carcinomas. However, it is to be noted as mentioned in the previous sections that PMA of the skin and of the eyelids is rare and majority of the lesions seen in clinical practice are metastasis from the breast, gastrointestinal tract, salivary glands, lacrimal glands, paranasal sinuses, lungs or kidneys, and ovary. Of particular interest are lesions from the breast and colon as they mimic PMA of the skin in pathological appearance [15]. Studies have shown that they stain positive for CK7 and are CK20 negative, similar to breast lesions. Whereas, lesions from the digestive tract are CK7 negative and CK20 positive [6, 12]. Although, breast lesions have similar results, skin metastasis from the breast is also uncommon with an incidence of 30% in metastatic breast cancer patients. Regardless, it is prudent for any patient with PMA to have a complete oncological and imaging workup for any occult malignancy as in our patient [3, 15]. PMA has shown positivity for several other immunostains in various reports like P63, carcinoembryonic antigen, epithelial membrane antigen, gross cystic disease fluid protein 15, low molecular weight cytokeratin, alpha lactalbumin, salivary type amylase, beta 2 microglobulin, thyroid transcription factor 1, thyroid transcription factor 3, estrogen and progesterone receptors. The significance of most of the stains is unknown. The utility of P63 in PMA remains controversial [1, 3]. P63 stains positive in myoepithelial cells and positivity is an indicator of an in situ origin from the skin [3, 16]. Qureshi et al. in their study of seven cases of PMA found that five out of seven cases were positive for markers of in situ component like P63 and concluded that it may aid in excluding metastasis from primaries like the breast [16]. However, reports have suggested that P63 is occasionally positive in cutaneous metastasis form the breast and lung. In Kanitakis and Chouvet‘s report of 45 cutaneous metastasis, P63 was positive in 11% of the cases. Given the literature available so far, it can be concluded that a combination of surgical pathology analysis with immunostaining and systemic workup for occult malignancy is needed for the diagnosis [3, 17].

Excision of the lesion, preferably with frozen section control of margins or Mohs micrographic surgery, is the treatment strategy preferred nowadays [3, 18]. Recurrence has always been a concern with PMA of the eyelid, with local recurrence rates of up to 40% following traditional wide excision [18]. In a report in which follow up of 26 cases was done for an average period of 26.3 months, the recurrence rate was 26% [3]. However, on analyzing 14 cases treated with Mohs micrographic surgery or frozen section control, the local recurrence rate was only 1%. Although Mohs micrographic surgery seems to have clear benefit in several individual reports and small analysis, larger studies are needed to establish the same [3, 19]. Given that very late recurrences have been reported, regular follow up over a prolonged period of time is essential [18-19]. The patient in our case was routinely followed up every 6-8 months. 

Table 1 represents the comparison of recent cases of PMA of the eyelid of which African American cases (2008-present) and Others (2013-present) are summarized. The immunohistochemistry profile, treatment, and recurrence have been shown.

**Table 1:** Comparison of recent cases of PMA of the eyelid; African American cases (2008-present), Others (2013-present). PMA, primary mucinous adenocarcinoma; CK7, cytokeratin 7; CK20, cytokeratin 20; ER, estrogen receptor; PR, progesterone receptor; GCDP-15, gross cystic disease fluid protein-15; NA, not analyzed.

	Case	Age	Sex	Race	Location	Immunohistochemistry	Treatment	Recurrence
1	Our case	62	F	African American	Left upper eyelid	CK7 + CK20 - P63 +	Excision with wide margins	Disease free for two years
2	Burris et al. [20]	71	F	Unknown	Left upper eyelid	CK7 + CK20 - P63 +	Mohs assisted by alcian blue staining	Unknown
3	Smith et al. [14]	89	M	Unknown	Right lower eyelid and canthus	Not done	Simple local tissue rearrangement	Unknown
4	Albasri et al. [18]	60	M	Unknown	Left lower eyelid	CK7 + CK 20 - P63 + CK 5/6 + ER + PR +	Wide local excision	Disease free for one year
5	Sanft et al. [8]	67	M	Unknown	Right lower eyelid	CK7 + CK 20 - P63 + GCDP-15 +	Excision	Recurrence after five years
6	Sanft et al. [8]	73	M		Right upper eyelid	CK7 + CK 20 - P63 NA GCDP-15 +	Excision	Unknown
7	Sanft et al. [8]	62	M	Unknown	Right lower eyelid	CK7 + CK 20 - P63 NA GCDP-15 +	Excision	Unknown
8	Chavez et al. [19]	50	M	Caucasian	Right upper eyelid	Not done	Mohs micrographic surgery	Disease free for three years
9	Burris et al. [1]	53	M	African American	Right lateral canthus	CK7 + CK 20 - P63 NA ER +	Local resection	Six recurrences between 1993 and 2010
10	Kalebi and Hale [4]	68	M	African American	Supraorbital area	CK7 + CK 20 - P63 NA GCDP-15 + Positive psammoma bodies	Excision	Unknown

## Conclusions

The PMA of the eyelid is uncommon, especially in females and the African American population. Histopathology and immunohistochemistry play a very important role in its diagnosis and to differentiate it from a metastatic lesion. Given the uncommon nature of this tumor and lack of data with regard to management, more literature especially pertaining to long-term follow up would help clinicians.
